# Simultaneous Burr and Cut Interruption Detection during Laser Cutting with Neural Networks

**DOI:** 10.3390/s21175831

**Published:** 2021-08-30

**Authors:** Benedikt Adelmann, Ralf Hellmann

**Affiliations:** Applied Laser and Photonics Group, Faculty of Engineering, University of Applied Sciences Aschaffenburg, Wuerzburger Straße 45, 63739 Aschaffenburg, Germany; ralf.hellmann@th-ab.de

**Keywords:** laser cutting, quality monitoring, artificial neural network, burr formation, cut interruption, fiber laser

## Abstract

In this contribution, we compare basic neural networks with convolutional neural networks for cut failure classification during fiber laser cutting. The experiments are performed by cutting thin electrical sheets with a 500 W single-mode fiber laser while taking coaxial camera images for the classification. The quality is grouped in the categories good cut, cuts with burr formation and cut interruptions. Indeed, our results reveal that both cut failures can be detected with one system. Independent of the neural network design and size, a minimum classification accuracy of 92.8% is achieved, which could be increased with more complex networks to 95.8%. Thus, convolutional neural networks reveal a slight performance advantage over basic neural networks, which yet is accompanied by a higher calculation time, which nevertheless is still below 2 ms. In a separated examination, cut interruptions can be detected with much higher accuracy as compared to burr formation. Overall, the results reveal the possibility to detect burr formations and cut interruptions during laser cutting simultaneously with high accuracy, as being desirable for industrial applications.

## 1. Introduction

Laser cutting of thin metal sheets using fiber or disk lasers is now a customary process in the metal industry. The key advantages of laser cutting are high productivity and flexibility, good edge quality and the option for easy process automation. Especially for highly automated unmanned machines, seamlessly combined in line with bending, separation or welding machines, a permanent high cut quality is essential to avoid material waste, downtime or damaging subsequent machine steps in mechanized process chains. As a consequence, besides optimizing the cutting machine in order to reduce the influence of disturbance variables, cut quality monitoring is also of utmost interest. 

The most common and disruptive quality defects are cut interruptions and burr formation [1]. To obtain high-quality cuts, process parameters, such as laser power, feed rate, gas pressure, working distance of the nozzle and focus position, respectively, are to be set appropriately. Imprecise process parameters and typical disturbance values like thermal lenses, unclean optics, damaged gas nozzles, gas pressure fluctuations and the variations of material properties may lead to cut poor-quality and, thus, nonconforming products. To ensure a high quality, an online quality monitoring system, which can detect multiple defects, would be the best choice in order to respond quickly and reduce downtime, material waste or cost-extensive rework. Until now, most reviewed sensor systems for monitoring laser cutting focus only on one single fault.

For detecting burr formation during laser cutting, different approaches using cameras, phododiodes or acoustic emission were investigated. In [2,3] burr formation, roughness and striation angle during laser cutting with a 6 kW CO_2_ laser are determined by using a NIR camera sampling with 40 Hz. By using two cameras in [4], laser cutting with a CO_2_ laser is monitored by observing the spark trajectories underneath the sheet and melt bath geometries and correlate this to the burr formation or overburning defects. A novel approach is used in [5], employing a convolutional neural network to calculate burr formation from camera images with a high accuracy of 92%. By evaluating the thermal radiation of the process zone with photodiodes [6], the burr height during fiber laser cutting can be measured from the standard deviation of a filtered photodiode signal. Results by using photodiode-based sensors integrated in the cutting head [7] showed that the mean photodiode’s current increases with lower cut qualities, while similar experiments revealed increasing mean photodiode currents at lower cut surface roughness [8]. An acoustic approach was investigated by monitoring the acoustic emission during laser cutting and deducing burr formation by evaluating the acoustic bursts [9]. 

Also for cut interruption detection, most approaches are based on photodiode signals or camera images. Photodiode-based methods for cut interruption detection are signal threshold-based [10], done by the comparison of different photodiodes [11] or based on cross-correlations [12]. However, all those methods have the disadvantage of requiring thresholds that vary with the sheet thickness or laser parameters. In addition, an adaptation to other materials or sheet thicknesses requires a large engineering effort to define respective threshold values by extensive investigations. To avoid this problem, [13] uses a convolutional neural network to calculate cut interruptions from camera images during fiber laser cutting of different sheet thicknesses with an accuracy of 99.9%. Another approach is performed by using a regression model based on polynomial logistics [14] to calculate the interruptions from laser machine parameters only. 

This literature review reveals that for both burr formation monitoring and cut interruption, individual detection schemes have previously been reported, but a combined and simultaneous detection for both failure patterns has not been reported so far. In addition, many of the previous studies applied CO_2_ lasers, which are often replaced nowadays by modern fiber or disk lasers, for which, in turn, fewer reports are available. To detect both failures with the same system, we chose the evaluation of camera images with neural networks, as they are able to achieve a high accuracy in detecting both cut failures [5,13]. The use of neural networks, especially for convolutional neural networks (CNN), has been demonstrated for various image classification purposes, such as face recognition and object detection [15,16], in medicine for cancer detection [17] and electroencephalogram (EEG) evaluations [18] or in geology for earthquake detection [19]. For failure analyses in technical processes, neural networks have also been successfully used for, e.g., concrete crack detection [20], road crack detection [21] or detecting wafer error determinations [22]. In addition, detecting different failure types with the same system has been successfully proven with neural networks, such as detecting various wood veneer surface defects [23] or different welding defects [24] during laser welding. 

The objective of this publication is to detect both burr formation and cut interruptions during single-mode laser cutting of electrical sheets from camera images with neural networks. The advantages of our system are, firstly, easy adaption to industrial cutting heads, which often already have a camera interface. Secondly, images are taken coaxially to the laser beam and are therefore independent of the laser cut direction. Thirdly, due to the use of a learning system the engineering effort is low when the system has to be adapted to other materials or sheet thicknesses. Two different neural network types are used, namely a basic neural network and a convolutional neural network. The basic neural network is faster and can detect bright or dark zones but is less able to extract abstractions of 2D features and needs a lot of parameters when the networks get more complex. On the other hand, convolutional neural networks are much better in learning and extracting abstractions of 2D features and usually need fewer parameters. However, they require a higher calculation effort due to many multiplications in the convolution layers [25,26].

The cutting of electrical sheets is chosen because it is an established process in the production and prototyping of electric motors and transformers [27,28,29,30], i.e., it is a relevant and contributing process step to foster e-mobility. In order to reduce the electrical losses caused by eddy currents, the rotor is assembled of a stack of thin electrical sheets with electrical isolation layers in between the sheets. The sheet thickness varies typically between 0.35 mm to 0.5 mm, with the eddy currents being lower for thinner sheets. As a result, for an electric motor, a large number of sheets with high quality requirements are necessary. Especially burr formations result in gaps between sheets or the burr can pierce the electrical isolation layer and connect the sheets electrically which both reduce the performance of motors drastically. Therefore, quality monitoring during laser cutting is of great interest for industrial applications.

## 2. Experimental

### 2.1. Laser System and Cutting Setup

In this study, a continuous wave 500 W single-mode fiber laser (IPG Photonics, Burbach, Germany) is used to perform the experiments. The laser system is equipped with linear stages (X, Y) for positioning the workpiece (Aerotech, Pittsburgh, PA, USA) and a fine cutting head (Precitec, Gaggenau, Germany) is attached to a third linear drive (Z). The assisted gas nitrogen with purity greater than 99.999% flows coaxially to the laser beam. The gas nozzle has a diameter of 0.8 mm and its distance to the workpiece is positioned by a capacitive closed loop control of the z-linear drive. The emitting wavelength of the laser is specified to be 1070 nm in conjunction with a beam propagation factor of M^2^ < 1:1. The raw beam diameter of 7.25 mm is focused by a lens with a focal length of 50 mm. The according Rayleigh length is calculated to 70 µm and the focus diameter to 10 µm, respectively.

**Figure 1:**
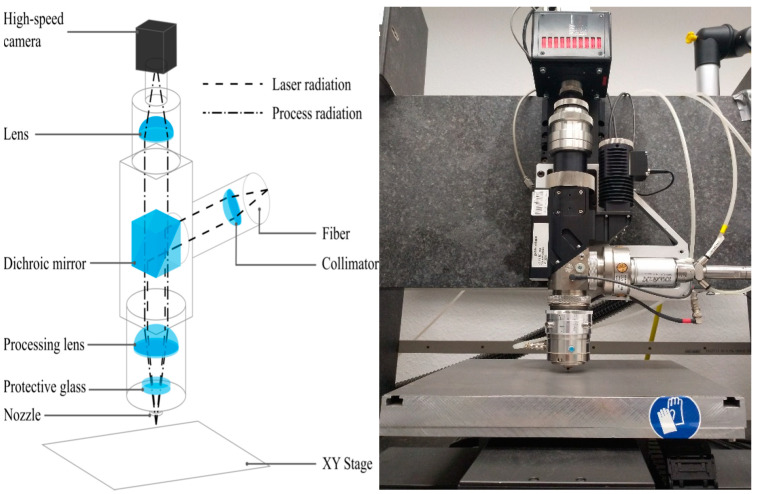
Optical setup of the cutting head (**left**) and image of the system (**right**).

The design of the cutting head with the high-speed camera and a photo of the laser system are illustrated in Figure 1. The dashed lines depict the primary laser radiation from the fiber laser, which is collimated by a collimator and reflected by a dichroic mirror downwards to the processing zone. There the laser radiation is focused by the processing lens through the protective glass onto the work piece, which is placed on the XY stages. The process radiation from the sheet radiates omnidirectional (dash-doted line), thus partly through the nozzle and protective glass and is collimated by the processing lens upwards. The process radiation passes the dichroic mirror and is focused by a lens onto the high-speed camera. The focus of the camera is set to the bottom side of the sheet in order to have a sharp view of possible burr formations.

### 2.2. Laser Cutting

The laser cuts are performed in electrical sheets of the type M270 (according to EN 10106 this denotes a loss of 2.7 W/kg during reversal of magnetism at 50 Hz and 1.5 T) with a sheet thickness of 0.35 mm. This sheet thickness is chosen because it fits well to the laser focus properties e.g., Rayleigh length and it is one of the most often used sheet thicknesses for electrical motors and transformers, because it provides a good compromise between low eddy currents and high productivity. Stacks of thicker sheets are faster to produce because less sheets are required per stack but with increasing sheet thickness also unwanted eddy currents increase. Thinner sheet thicknesses require a higher production effort per stack and are more difficult to cut because they are very flexible, and warp under the gas pressure and thermal influence. In these experiments only one sheet thickness is used, but please note that in previous publications with similar systems an adaptation of the results to other sheet thicknesses was possible with only minor additional expenses [5,13].

As ad-hoc pre-experiments reveal, the parameter combination of a good quality cut is a laser power of 500 W, a feed rate of 400 mm/s and a laser focus position on the bottom side of the metal sheet. The gas nozzle has a diameter of 0.8 mm and is paced 0.5 mm above the sheet surface and the gas pressure is 7 bar. For the experimental design, the parameters are varied to intentionally enforce cut failures. Burr formations are caused by less gas flow into the cut kerf due to higher nozzle to sheet distance, lower gas pressure, an overvalued power to feed rate ratio or damaged nozzles. Cut interruptions are enforced by too high feed rates or too low laser power.

**Figure 2:**
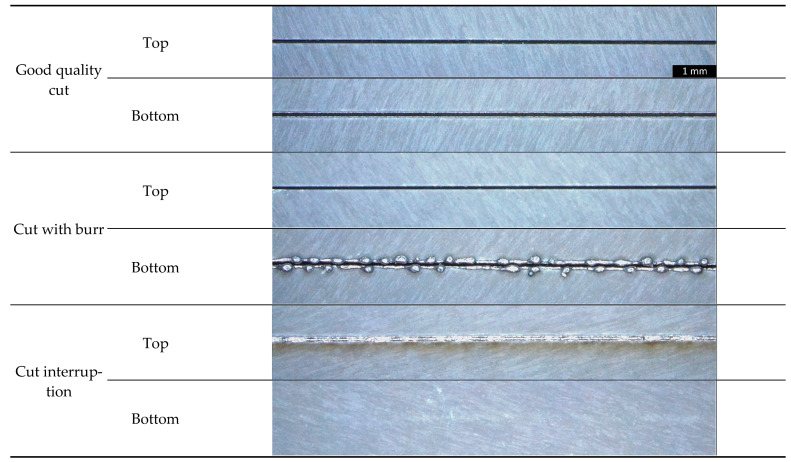
Images of the top and bottom side of laser cuts with and without cut errors taken with an optical microscope after laser cutting.

In the experimental design, 39 cuts with different laser parameters are performed for training the neural network and 22 cuts are performed for testing, with the cuts being evenly distributed to the three cut categories (good cut, cuts with burr formation and cut interruptions). A table of all cuts with laser machine parameters, category and use can be found in the Appendix A. The cuts are designed from a straight line including acceleration and deceleration paths of the linear stages. Exemplifying images of the sheets from all three cut categories taken by optical microscope after the cutting process are shown in Figure 2. Firstly, for a good quality cut, both top and bottom side of the cut kerf are characterized by clear edges without damages. Secondly, for a cut with burr, the top side is similar to the good quality cut, however on the bottom side drops of burr formation are clearly visible. Thirdly, the images of the cut interruption reveal a molten line on the sheet top side and only a slightly discolored stripe on the bottom side with both sides of the sheet not being separated. 

### 2.3. Camera and Image Acquisition

For image acquisition during laser cutting, we used a high-speed camera (Fastcam AX50, Photron, Tokyo, Japan) with a maximum frame rate of 170,000 frames per second. The maximum resolution is 1024 × 1024 pixels, with a square pixel size of 20 × 20 μm^2^ in combination with a Bayer CFA Color Matrix. For process image acquisition, videos of the laser cutting process are grabbed with a frame rate of 10 kilo frames per second with an exposure time of 2 µs and a resolution of 128 × 64 pixels. Even at this high frame rate, no oversampling occurs and consecutive images are not similar, because the relevant underlying melt flow dynamics are characterized by high melt flow velocities in the range of 10 m/s [31] and vary therefore at estimated frequencies between 100 kHz and 300 kHz [32]. Please note, due to the lack of external illumination in the cutting head, the brightness in the images are caused by the thermal radiation of the process zone. 

**Figure 3:**
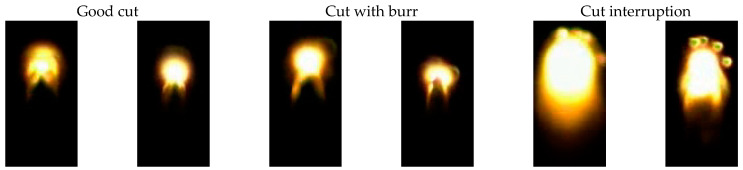
Examples of camera images of the three cut categories taken during laser cutting with the high speed camera.

Two exemplifying images of each cut category are shown in Figure 3 with the cut direction always upwards. The orientation of the images is always the same because the straight lines are cut in the same direction. For complex cuts, images with the same orientation can be transformed from various oriented images by rotation based on the movement direction of the drives. In these images, brightness is caused by the thermal radiation of the hot melt. Good cuts are characterized by a bright circle at the position on the laser focus, and below this, two tapered stripes indicating the flowing melt at the side walls of the cut kerf, because in the middle the melt bath is blown out first. The cuts with burr are similar to the good quality cuts but tapered stripes are formed differently. The cut interruptions are very different to the other categories and are characterized by larger bright areas and a more elliptical shape with no tapered stripes.

From the 39 laser cuts, the experimental design delivers the same number of training videos with overall 52 thousand training images, while from the 22 testing cuts 34 thousand test images are provided. It is worth to mention, that the size of several ten thousands of training images is typical for training neural networks [33]. For both training and testing, the images are almost evenly distributed on the three categories with cut interruptions being slightly underrepresented. The reason for this underrepresentation is, that cut interruptions only occur at high feed rates, i.e., images from acceleration and deceleration paths can be used only partially and, in turn, less images per video can be captured.

### 2.4. Computer Hardware and Neural Network Design

For learning and evaluating the neural network, a computer with an Intel Core 7-8700 processor with a 3.2-GHz clock rate in combination with 16-GB DDR4 RAM was used. All calculations are performed with the CPU rather than the GPU to show that the machine learning steps are also possible to run on standard computers, which are usually integrated with laser cutting machines. The used software was TensorFlow version 2.0.0 in combination with Keras version 2.2.4 (Software available from: https://tensorflow.org (accessed on 24 March 2021)).

**Figure 4:**
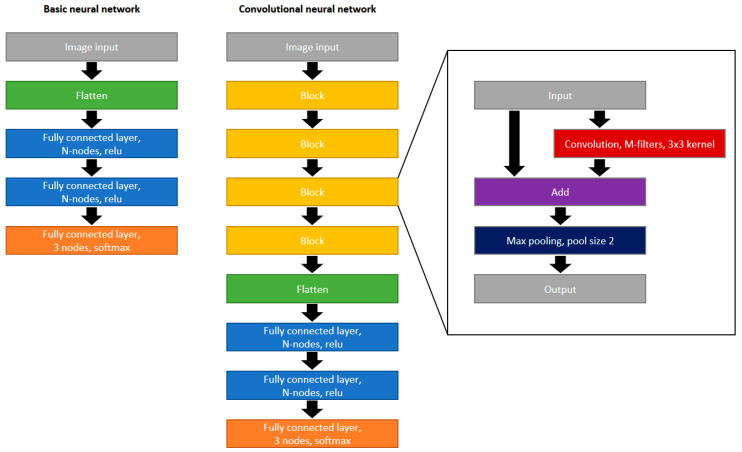
Design of the two neural networks.

In most publications about image classification with neural networks, the images have major differences. In contrast, in the images captured in our experiments, the object to analyze always has the same size, orientation and illumination conditions which should simplify the classification when compared to classifying common, moving items like vehicles or animals [34,35]. Furthermore, our images have a rectangular shape with 128 × 64 pixels, while most classification algorithms are optimized on square images sizes having mostly a resolution of 224 × 224 pixels like MobileNet, SqueezeNet or AlexNet [36,37]. Because an enlargement of the image size slows the system drastically, two self-designed and completely different neural network are used with many elements being adapted to other, often used neural networks. The first network, as shown in Figure 4 is a basic network without convolution and only consists of image flattening followed by two fully connected layers with N nodes and ReLU Activation. To classify the three different cut categories, a fully connected layer with 3 nodes and softmax activation completes the network. The second network is a convolutional neural network with four convolution blocks followed by the same three fully connected layers as in the basic network. Each block consists of a convolution layer with a kernel size of 3 × 3 and M filters, which the output of the convolution is added with input of the block. Such bypasses are most common in, e.g., MobileNet [36]. To reduce the number of parameters, a max pooling layer with a common pool size of 2 × 2 is used [26]. In contrast to often neural networks used in the literature, we use a constant instead of an increasing filter number for subsequent convolution layers and we use normal convolutions rather than separable or pointwise convolutions. Because every block halves the image size in 2 dimensions, after 4 blocks the image size is 8 × 4 × M. The fully connected layers after the flattening have the same number of nodes as the number of parameters delivered by the flattened layer. The used model optimizer is Adam, which according to [38], together with SDG (Stochastic Gradient Descent) provides superior optimization results. Furthermore, we use the loss function “categorical crossentropy” to enable categorical outputs (one hot encoding), and the metrics “accuracy”. 

### 2.5. Methodology

**Figure 5:**
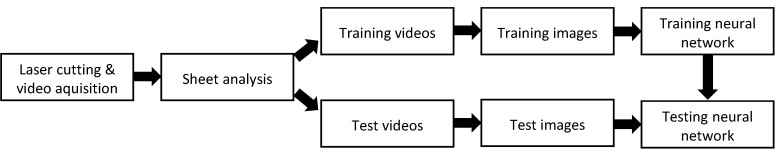
Workflow diagram.

The methodology of our experiments is shown in the workflow diagram in Figure 5. In a first step, the laser cuts are performed and during cutting videos are taken from the process zone with the high speed camera, some of these images have been shown in Figure 3. After cutting, the cut kerfs are analyzed by with an optical microscope and categorized manually whether a good cut, burr formation or a cut interruption occurred (examples of these images shown in Figure 2). Based on this classification, the videos taken during laser cutting are labeled with the corresponding class. In case the cut quality changes within one cut, the video is divided, so the quality is constant within a video. Then the videos are separated in training videos and test videos, so the images for testing are not from videos used for training. From the training videos, the single frame is extracted and with these images the neural network is trained. Furthermore, the single frames are extracted from the test videos, and the resulting images are used to test the trained neural network. 

## 3. Results

### 3.1. Training Behaviour

**Figure 6:**
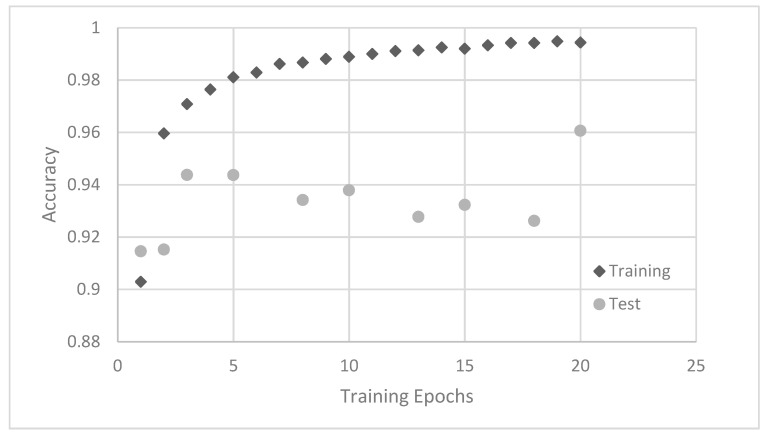
Training accuracy and test accuracy of a convolutional neural network with 16 filters.

The different neural networks are trained on the training dataset and the performance is calculated on the test dataset. Exemplarily, the training behavior of the convolutional neural network with 16 filters in each convolution is shown in Figure 6. Apparently, the training accuracy rises continuously with the training epochs, reaching 99% after 10 epochs and 99.5% after 20 epochs, respectively. On the other hand, the test accuracy reaches 94% after three epochs and fluctuates with further training around this level, which is a typical behavior for neural network training [39]. Even further training, above 20 epochs, results only in a fluctuation of the accuracy rather than a continuous increase. To reduce the deviation of the test results for comparisons between different networks, the mean of the test results between 10 and 20 epochs is used.

### 3.2. Basic Neural Network

**Figure 7:**
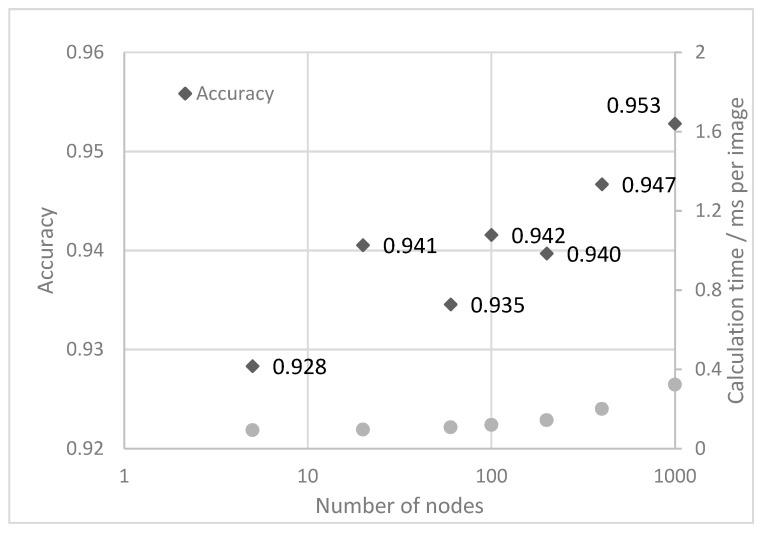
Accuracy of the basic neural network as a function of nodes per fully connected layer.

To determine the performance of the basic neural networks, those with node numbers N between 5 and 1000 are trained on the training dataset and tested on the test dataset. The mean test accuracy between 10 and 20 training epochs and the required calculation time per image are shown in Figure 7. It is obvious that the accuracy for a very small network with only five nodes is quite high, being 92.8%, and the calculation time of 0.1 ms per image being very fast. With an increasing number of nodes, the accuracy increases to a maximum of 95.2% at 1000 nodes, which is accompanied by a higher calculation time of 0.32 ms. Parallel to the calculation time, also the trainable parameters increase with the number of nodes starting from 122 thousand parameters for five nodes and reaching 25 million parameters at 1000 nodes. A further increase of the parameters is not considered to be useful, because the training dataset consist of 420 million of pixels (number of images x pixels per image), so the neural network tend to over fit the training dataset rather than developing generalized features. Generally, with the basic neural network accuracies of 94% (mean) are achievable.

### 3.3. Convolutional Neural Network

**Figure 8:**
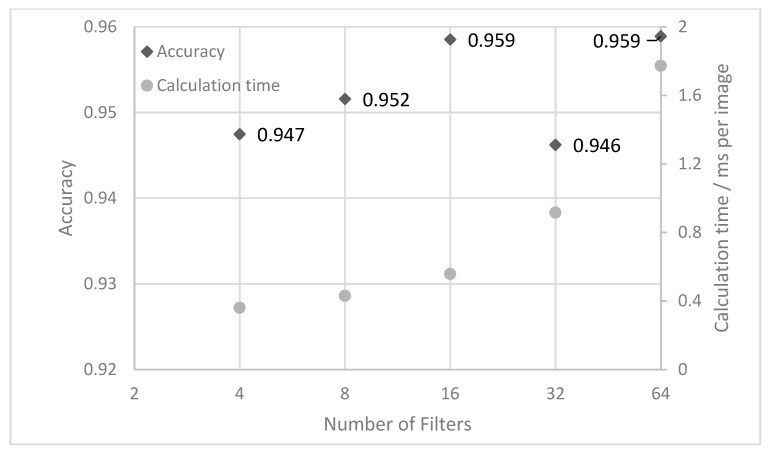
Test accuracy for the convolutional neural network as a function of the number of filters.

Under the same conditions as the basic neural network, the convolutional neural network is also trained and tested. The results of the accuracy and calculation time for filter numbers between 4 and 64 are depicted in Figure 8. The accuracy of the neural network is quite high for all filter numbers and fluctuates between 94.6% and 95.8% with no clear trend. In addition, the accuracy also varies for the same network when it is calculated several times. However, the calculation time increases clearly with the number of filters from 0.36 ms per image to 1.77 ms. The number of trainable parameters start with 34 thousand for four filters and increases to 8.4 Million for the 64 filters (details how to calculate the number of parameters are described in [25]). For the mean, the convolutional neural network is able to classify about 95% of the image correctly. 

### 3.4. Comparison between Cut Failures

**Figure 9:**
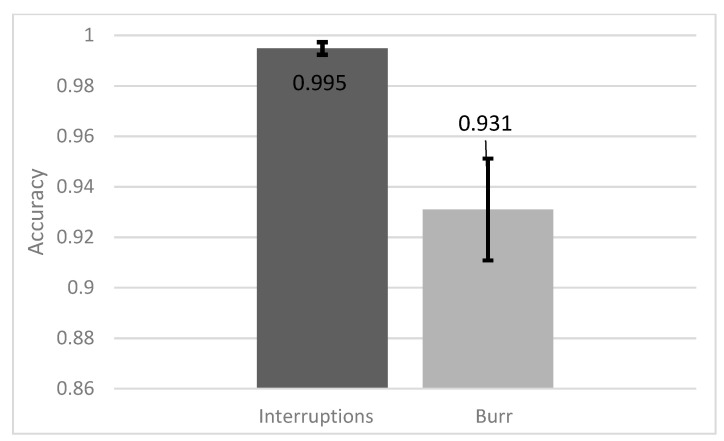
Comparison of the test accuracy between interruptions and burr formations.

Since the literature is available for both burr detection and cut interruptions during laser cutting, which vary strongly in accuracy, the performance of our neural networks in detecting one cut failure is determined. Therefore, the accuracy in classifying good cuts and cuts with burr as well as good cuts and cut interruptions is calculated separately. For this investigation, the convolutional neural network with 16 filters is chosen, because it provides high accuracy and a comparable moderate calculation time. The results of the separated classification are shown in Figure 9. It is obvious that the detection of cut interruptions is very reliable with the accuracy being 99.5%, as being compared to 93.1% when detecting burr formation. The reason for this can also be seen in Figure 3, where good cuts are much more similar to cuts with burr, while cut interruptions look very different to both of the other failure classes. Both values individually agree with the literature values, which are 99.9% for the cut interruptions [13] and 92% for the burr detection [5], yet for burr detection in the literature a more complex burr definition is chosen. This shows that cut interruptions are much easier to detect from camera images compared to burr formations.

### 3.5. Error Analysis

**Figure 10:**
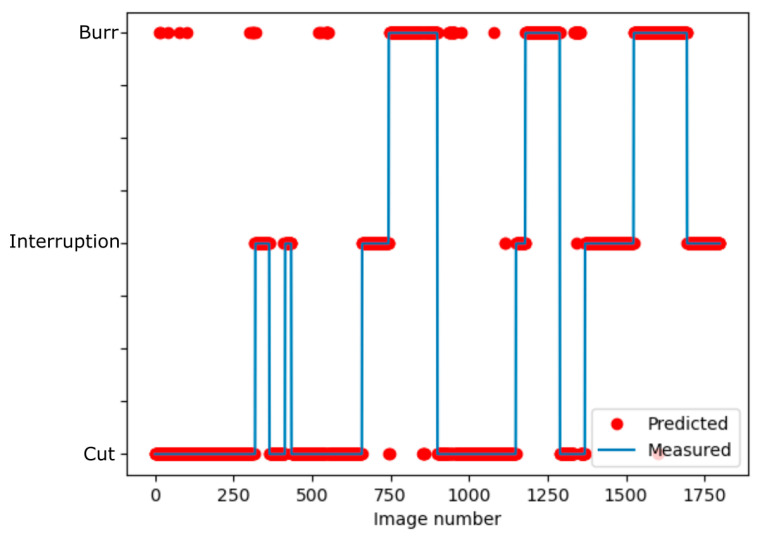
Measured image class and prediction by the neural network.

For the error analysis, single misclassified images and the distribution of misclassifications are analyzed. For the temporal distribution, a video of a cut with different cut qualities is produced. The measured quality obtained by the optical microscope and the prediction of the convolutional neural network with 16 filters is shown in Figure 10. Misclassifications are indicated by red dots that are not placed on the blue line and it can clearly be seen, which misclassifications occur more often than others. The most frequent misclassifications are cuts predicted as burr. Interruptions are rarely misclassified and other images are seldom misclassified as interruptions, which accompanies the results in Section 3.4. The distribution of the misclassifications reveals no concentration on a specific sector but minor accumulations of several misclassifications are observed. In addition, some areas without any misclassification or only single misclassifications can be found. These results reveal that misclassifications do not occur all at once or at a special event but are widely distributed.

**Figure 11:**
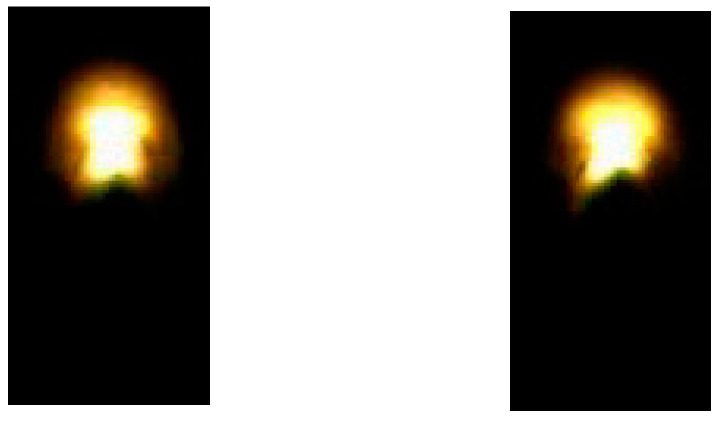
Two examples of cuts missclassified as burr.

To analyze the misclassified images, two exemplified images from a good cut classified as cut with burr are shown in Figure 11. In contrast to the images in Figure 3, where the bright area is followed by two tapered stripes, in Figure 11, these stripes are hardly observed. However, these following stripes are important for the classification, because in this area the burr is generated. Therefore, in the case of missing stripes, the classification between cuts with and without burr is difficult and thus characterized by many misclassifications.

## 4. Discussion

With the classification in three different categories during laser cutting, good cuts can be distinguished from cuts with burr and cut interruptions. The convolutional neural network has, depending on the number of filters, a better classification accuracy by about 1% when compared to the basic networks. The maximum accuracy for the basic neural networks (1000 nodes) is also lower, being 95.2% as compared to a 95.8% accuracy of the convolutional neural network with 16 filters. Nevertheless, the difference between both neural network types is small, which can be explained by the objects in the images always having the same size, orientation and brightness, which is not usually the case for many other classification tasks [34,35]. As a consequence, the basic neural network can classify the images by bright or dark zones and does not necessarily require learning and extracting abstractions of 2D features which is the main advantage of convolutional neural networks [25,26].

For the required accuracy, the size of the cut failure has to be considered. Because of the accuracy being below 100%, a post algorithm is necessary which should report an error only when a certain amount of failures occurs in a sequent number of images. To detect geometrically long failures, which can occur, e.g., by unclean optics, our classification system is adequate. Very short failures, like single burr drops when cutting an edge, are probably not be detectable with our system. It is remarkable for the results with both neural networks, however, that at least 92.8% accuracy (cf. Figure 7) can be achieved with any network configuration independent from network type, number of nodes or filters. This means that about 93% of the images are easy to classify because they differ strongly between the categories. Furthermore, about 2% of the images can be classified by more complex neural networks (cf. Section 3.2 and Section 3.3). About 5% of the images, mostly between the categories good cuts and cuts with burr formation, are very difficult to classify because the images are quite similar (cf. Figure 3). For an industrial application, it has to be further considered whether the intentionally enforced cut failures are representative for typical industrial cut failures, e.g., as a result of unclean optics, which are not reproducible in scientific studies. 

The main advantage of the basic neural network is the much lower computation time between 0.1 ms and 0.32 ms, while the convolutional neural network requires 0.36 ms to 1.7 ms, respectively. For typical available industrial cameras having maximum frame rates in the range of 1000 Hz, a calculation time for the classification of about 1 ms is sufficient, which is fulfilled by all basic and most of our convolutional neural networks. A similar frame rate was also used by [5] when detecting burr formations during laser cutting. With maximum cutting speeds of modern laser machines in the range of 1000 mm/s still a local resolution of 1 mm is achieved which can clearly be considered as adequate for industrial use. 

Following this fundamental and comparative analysis, future investigations have to address field trials of the proposed sensor system and classification scheme in industrial cutting processes. Within such industrial environments additional error sources may appear and further reduce the cut quality, such as damaged gas nozzles or partially unclean optics which in turn are difficult to reproduce under laboratory conditions. The images from these error sources can be added to the training data and improve the detection rate of the classification system. To improve the detection rate it is also possible to classify not a single image but a series of 3 to 10 subsequent images, which reduces the influence of a single misleading image. 

## 5. Conclusions

Overall, with our neural network approach, two cut failures during laser cutting can be detected simultaneously by evaluating camera images with artificial neural networks. With different neural network designs up to 95.8% classification accuracy can be achieved. Generally, convolutional neural networks have only minor classification advantages of about 1% over basic neural networks, while the basic neural networks are considerably faster in calculation. The detection of cut interruptions is remarkably higher when compared to the burr formation, because the images of cut interruptions are more different from the good cuts compared to the images with burr formation. In general, the detection rate is high enough to advance industrial applications. 

## Data Availability

Not applicable.

## Appendix A

Table of all performed laser cuts with machine parameters, cut category and use for training and test.

**Nr:**	**Laser**	**Feed Rate**	**Nozzle Distance**	**Focus Position**	**Category**	**Use**
	W	mm/s	mm	mm		
1	500	600	0.5	−1.25	Cut	Training
2	300	400	0.5	−1.25	Cut	Training
3	500	500	0.5	−1.25	Cut	Training
4	500	300	0.5	−1.25	Cut	Training
5	300	600	0.5	−1.25	Interruption	Test
6	200	500	1,0	−1.75	Interruption	Training
7	500	500	0.8	−1.55	Burr	Training
8	500	600	0.5	−1.25	Cut	Test
9	250	500	0.5	−1.25	Interruption	Test
10	500	400	0.5	−1.25	Cut	Test
11	500	600	0.5	−1.25	Interruption	Training
12	500	500	1,0	−1.75	Burr	Training
13	500	500	0.5	−1.25	Cut	Test
14	500	300	0.5	−1.25	Cut	Training
15	500	200	0.5	−1.25	Cut	Test
16	500	500	0.5	−1.25	Cut	Training
17	500	500	0.5	−1.25	Interruption	Test
18	400	500	0.9	−1.65	Burr	Training
19	500	500	0.8	−1.55	Burr	Training
20	200	500	1,0	−1.75	Interruption	Training
21	500	300	0.5	−1.45	Burr	Training
22	150	500	0.5	−1.25	Interruption	Test
23	500	400	0.5	−1.25	Cut	Training
24	500	500	0.5	−1.25	Cut	Test
25	500	400	0.5	−1.25		Training
26	400	500	0.8	−1.55	Burr	Training
27	500	500	0.8	−1.55	Burr	Test
28	150	500	0.5	−1.25	Interruption	Training
29	200	500	1,0	−1.75	Interruption	Test
30	400	500	0.9	−1.65	Burr	Training
31	300	600	0.5	−1.25	Interruption	Training
32	500	500	1,0	−1.75	Burr	Training
33	500	300	0.5	−1.25	Cut	Test
34	400	500	1,0	−1.75	Burr	Test
35	500	500	0.5	−1.25	Interruption	Training
36	500	400	0.5	−1.25	Cut	Training
37	150	500	0.5	−1.25	Interruption	Training
38	400	500	0.5	−1.45	Burr	Training
39	500	600	0.5	−1.25	Interruption	Training
40	400	400	0.5	−1.25	Cut	Training
41	500	600	0.5	−1.25	Cut	Training
42	500	600	0.5	−1.25	Interruption	Test
43	400	500	0.8	−1.55	Burr	Test
44	400	500	0.5	−1.45	Burr	Test
45	400	400	0.5	−1.25	Cut	Test
46	500	500	0.5	−1.25	Cut	Training
47	500	200	0.5	−1.25	Cut	Training
48	300	400	0.5	−1.25	Interruption	Training
49	400	500	0.5	−1.45	Burr	Training
50	500	400	0.5	−1.25	Cut	Test
51	500	500	0.8	−1.55	Burr	Training
52	400	500	0.9	−1.65	Burr	Training
53	400	500	0.9	−1.65	Burr	Test
54	500	300	0.5	−1.45	Burr	Training
55	300	400	0.5	−1.25	Cut	Training
56	500	300	0.5	−1.45	Burr	Test
57	250	500	0.5	−1.25	Interruption	Training
58	300	400	0.5	−1.25	Cut	Training
59	300	400	0.5	−1.25	Interruption	Training
60	300	600	0.5	−1.25	Interruption	Training
61	300	400	0.5	−1.25	Interruption	Test
